# 5-Butyl­amino-6-(4-fluoro­phen­yl)-7-oxo-1-*p*-tolyl-6,7-dihydro-1*H*-pyrazolo[4,3-*d*]pyrimidine-3-carbonitrile

**DOI:** 10.1107/S1600536808009744

**Published:** 2008-04-16

**Authors:** Ji-Huan Hu, Ming-Hu Wu

**Affiliations:** a College of Chemistry, Central China Normal University, Wuhan 430079, People’s Republic of China; b Department of Chemistry and Life Sciences, Xianning College, Xianning 437100, People’s Republic of China

## Abstract

In the title compound, C_23_H_21_FN_6_O, the dihedral angle between the fluoro­phenyl and pyrimidinone rings is 75.9 (1)°, and the dihedral angle between the methyl­phenyl and pyrazole rings is 40.3 (1)°. In the crystal structure, weak C—H⋯π(arene) and C—N⋯π(arene) inter­actions and intermolecular C—H⋯N and N—H⋯O hydrogen-bonding inter­actions are present.

## Related literature

For background information, see: Bell *et al.* (1991 ▶); Zhao *et al.* (2006 ▶); Allerton *et al.* (2006 ▶).
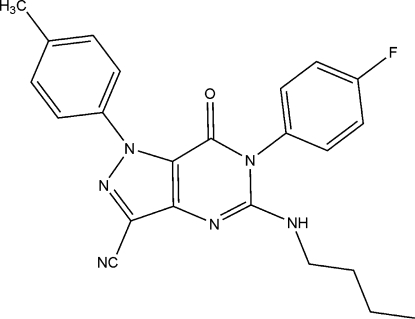


## Experimental

### 

#### Crystal data

C_23_H_21_FN_6_O
*M* *_r_* = 416.46
Monoclinic, 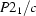

*a* = 11.8800 (5) Å
*b* = 9.36020 (4) Å
*c* = 19.0053 (8) Å
β = 91.178 (1)°
*V* = 2112.93 (13) Å^3^
*Z* = 4
Mo *K*α radiation
μ = 0.09 mm^−1^
*T* = 295 (2) K
0.30 × 0.20 × 0.20 mm

#### Data collection

Bruker SMART APEX CCD diffractometer
Absorption correction: multi-scan (*SADABS*; Sheldrick, 2001 ▶) *T*_min_ = 0.973, *T*_max_ = 0.982
12013 measured reflections
3704 independent reflections
3085 reflections with *I* > 2σ(*I*)
*R*_int_ = 0.031

#### Refinement

*R*[*F* ^2^ > 2σ(*F* ^2^)] = 0.055
*wR*(*F* ^2^) = 0.154
*S* = 1.11
3704 reflections
285 parameters
1 restraint
H atoms treated by a mixture of independent and constrained refinement
Δρ_max_ = 0.23 e Å^−3^
Δρ_min_ = −0.26 e Å^−3^

### 

Data collection: *SMART* (Bruker, 2001 ▶); cell refinement: *SAINT* (Bruker, 2001 ▶); data reduction: *SAINT*; program(s) used to solve structure: *SHELXS97* (Sheldrick, 2008 ▶); program(s) used to refine structure: *SHELXL97* (Sheldrick, 2008 ▶); molecular graphics: *PLATON* (Spek, 2003 ▶); software used to prepare material for publication: *PLATON*.

## Supplementary Material

Crystal structure: contains datablocks global, I. DOI: 10.1107/S1600536808009744/er2051sup1.cif

Structure factors: contains datablocks I. DOI: 10.1107/S1600536808009744/er2051Isup2.hkl

Additional supplementary materials:  crystallographic information; 3D view; checkCIF report

## Figures and Tables

**Table 1:** Hydrogen-bond geometry (Å, °) *Cg*1 is the centroid of the C14–C19 ring and *Cg*2 is the centroid of the N4/C9/C10/C13/N5/C12 ring.

*D*—H⋯*A*	*D*—H	H⋯*A*	*D*⋯*A*	*D*—H⋯*A*
N6—H6*A*⋯O1^i^	0.861 (10)	2.32 (2)	2.904 (2)	125 (2)
C15—H15⋯N3^ii^	0.93	2.58	3.449 (3)	155
C19—H19⋯N3^iii^	0.93	2.50	3.219 (3)	134
C6—H6⋯*Cg*1^iv^	0.93	2.82	3.671 (2)	152
C11—N3⋯*Cg*2^v^	1.141 (3)	3.621 (3)	3.710 (3)	85.4 (2)

Symmetry codes: (i) 
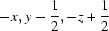
; (ii) 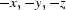; (iii) 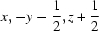; (iv) 
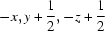
; (v) 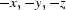.

**Fig. 1.**
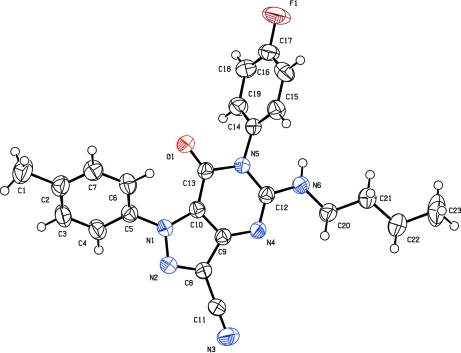
View of the molecule with the atom-labeling scheme. Displacement ellipsoids are drawn at the 50% probability level.

**Fig. 2.**
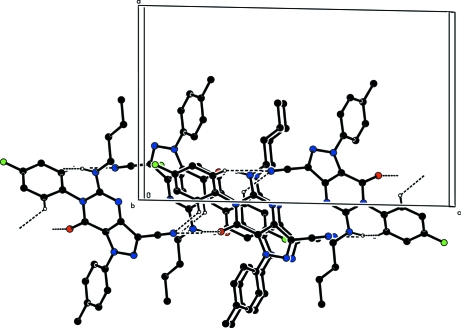
The packing viewed down the b axis. Intermolecular hydrogen bonds are shown as dashed lines.

## supplementary crystallographic information

### Comment

Pyrazolo[4,3-d]pyrimidin-7-ones have been reported as potent and selective inhibitors of PDE5 (Bell *et al.*, 1991; Zhao *et al.*, 2006). Sildenafil citrate, a pyrazolo[4,3-d]pyrimidin-7-one derivative, is the first orally effective PDE5 inhibitor approved for the treatment of erectile dysfunction. Its advent has spurred significant interest in the development of additional PDE5 inhibitors (Allerton *et al.*, 2006). Herein, the title compound as Sildenafil analog was synthesized and determined by X-ray crystal diffraction in order to find new potent PDE5 inhibitors.

In the molecule (Fig. 1), the dihedral angle between the fluorophenyl and pyrimidinon ring is 75.9 (1)°, and the dihedral angle between the methylphenyl and pyrazole ring is 40.3 (1)°. The atoms O1, N1–N6, C5, C8–C14 and C20 are nearly coplanar, and N6, C21–C23 formed a plane.

In the crystal structure, molecules are linked by weak C—H···π (arene) interactions, which connected H6 to the centroid of atoms C14–19, *Cg*1, (symmetry code: -*x*,1/2 + *y*,1/2 - *z*). In addition, the crystal structure is stablized by intermolecular N—H···O and intramolecular C—H···N hydrogen-bonding interactions (Fig. 2).

### Experimental

To a solution of 4-(3-cyano-5-ethoxycarbonyl-*p*-tolyl-1*H*-pyrazolyl) iminophosphorane (1.59 g, 3 mmol) in absolute anhydrous CH_2_Cl_2_, 4-fluorophenylisocyanate (3 mmol) was added at room temperature. The reaction mixture was left unstirred for 6 h at 273–278 K, whereafter a slight excess of butylamine (3.1 mmol) was added. After that, the reaction mixture was stirred for 6 h, the solution was cooled and the reaction product was recrystallized from EtOH and CH_2_Cl_2_ to give colorless crystals of the title compound in yield 93%, suitable for X-ray analysis.

### Refinement

All H atoms bound to C atoms were placed in calculated positions (C—H = 0.93–0.97 Å) and included in the riding model approximation, with *U*_iso_(H) = 1.2*U*_eq_(C) or 1.5*U*_eq_(methyl C).

### Crystal data

**Table:**

C_23_H_21_FN_6_O	*F*_000_ = 872
*M**_r_* = 416.46	*D*_x_ = 1.309 Mg m^−^^3^
Monoclinic, *P*2_1_/*c*	Mo *K*α radiation λ = 0.71073 Å
Hall symbol: -P 2ybc	Cell parameters from 3510 reflections
*a* = 11.8800 (5) Å	θ = 2.4–23.9º
*b* = 9.36020 (4) Å	µ = 0.09 mm^−^^1^
*c* = 19.0053 (8) Å	*T* = 295 (2) K
β = 91.1780 (10)º	Block, colourless
*V* = 2112.93 (13) Å^3^	0.30 × 0.20 × 0.20 mm
*Z* = 4	

### Data collection

**Table:**

Bruker SMART APEX CCD diffractometer	3704 independent reflections
Radiation source: fine-focus sealed tube	3085 reflections with *I* > 2σ(*I*)
Monochromator: graphite	*R*_int_ = 0.031
*T* = 295(2) K	θ_max_ = 25.0º
φ and ω scans	θ_min_ = 2.1º
Absorption correction: multi-scan(SADABS; Sheldrick, 2001)	*h* = −14→14
*T*_min_ = 0.973, *T*_max_ = 0.982	*k* = −11→11
12013 measured reflections	*l* = −16→22

### Refinement

**Table:**

Refinement on *F*^2^	Secondary atom site location: difference Fourier map
Least-squares matrix: full	Hydrogen site location: inferred from neighbouring sites
*R*[*F*^2^ > 2σ(*F*^2^)] = 0.055	H atoms treated by a mixture of independent and constrained refinement
*wR*(*F*^2^) = 0.154	*w* = 1/[σ^2^(*F*_o_^2^) + (0.0811*P*)^2^ + 0.4221*P*] where *P* = (*F*_o_^2^ + 2*F*_c_^2^)/3
*S* = 1.11	(Δ/σ)_max_ < 0.001
3704 reflections	Δρ_max_ = 0.23 e Å^−^^3^
285 parameters	Δρ_min_ = −0.26 e Å^−^^3^
1 restraint	Extinction correction: none
Primary atom site location: structure-invariant direct methods	

### Special details

**Table:**

Geometry. All e.s.d.'s (except the e.s.d. in the dihedral angle between two l.s. planes) are estimated using the full covariance matrix. The cell e.s.d.'s are taken into account individually in the estimation of e.s.d.'s in distances, angles and torsion angles; correlations between e.s.d.'s in cell parameters are only used when they are defined by crystal symmetry. An approximate (isotropic) treatment of cell e.s.d.'s is used for estimating e.s.d.'s involving l.s. planes.
Refinement. Refinement of *F*^2^ against ALL reflections. The weighted *R*-factor *wR* and goodness of fit *S* are based on *F*^2^, conventional *R*-factors *R* are based on *F*, with *F* set to zero for negative *F*^2^. The threshold expression of *F*^2^ > σ(*F*^2^) is used only for calculating *R*-factors(gt) *etc*. and is not relevant to the choice of reflections for refinement. *R*-factors based on *F*^2^ are statistically about twice as large as those based on *F*, and *R*-factors based on ALL data will be even larger.

### Fractional atomic coordinates and isotropic or equivalent isotropic displacement parameters (Å^2^)

**Table:**

	*x*	*y*	*z*	*U*_iso_*/*U*_eq_	
C1	0.6274 (2)	0.3875 (3)	0.22154 (16)	0.0778 (9)	
H1A	0.6429	0.3528	0.2683	0.117*	
H1B	0.6068	0.4865	0.2236	0.117*	
H1C	0.6934	0.3769	0.1937	0.117*	
C2	0.53165 (19)	0.3027 (3)	0.18861 (12)	0.0517 (6)	
C3	0.55219 (19)	0.1809 (3)	0.15018 (14)	0.0614 (7)	
H3	0.6261	0.1512	0.1443	0.074*	
C4	0.46540 (18)	0.1021 (3)	0.12017 (14)	0.0552 (6)	
H4	0.4809	0.0204	0.0942	0.066*	
C5	0.35615 (17)	0.1450 (2)	0.12887 (11)	0.0399 (5)	
C6	0.33266 (18)	0.2674 (2)	0.16595 (12)	0.0473 (6)	
H6	0.2587	0.2978	0.1708	0.057*	
C7	0.4207 (2)	0.3443 (2)	0.19588 (13)	0.0525 (6)	
H7	0.4049	0.4262	0.2216	0.063*	
C8	0.18880 (17)	−0.0696 (2)	0.01909 (11)	0.0406 (5)	
C9	0.11084 (16)	−0.0625 (2)	0.07421 (10)	0.0362 (5)	
C10	0.16433 (16)	0.0224 (2)	0.12452 (11)	0.0361 (5)	
C11	0.17644 (18)	−0.1440 (3)	−0.04584 (12)	0.0468 (6)	
C12	−0.03810 (16)	−0.1084 (2)	0.14132 (11)	0.0371 (5)	
C13	0.11533 (16)	0.0439 (2)	0.19173 (11)	0.0368 (5)	
C14	−0.04608 (16)	−0.0095 (2)	0.26283 (10)	0.0378 (5)	
C15	−0.13358 (18)	0.0858 (2)	0.26853 (12)	0.0465 (5)	
H15	−0.1606	0.1347	0.2291	0.056*	
C16	−0.1812 (2)	0.1083 (3)	0.33376 (13)	0.0533 (6)	
H16	−0.2413	0.1709	0.3384	0.064*	
C17	−0.1383 (2)	0.0369 (3)	0.39086 (12)	0.0544 (6)	
C18	−0.0514 (2)	−0.0580 (3)	0.38634 (12)	0.0555 (6)	
H18	−0.0241	−0.1056	0.4261	0.067*	
C19	−0.00515 (19)	−0.0814 (2)	0.32127 (12)	0.0481 (6)	
H19	0.0538	−0.1459	0.3168	0.058*	
C20	−0.19690 (18)	−0.2591 (2)	0.10305 (12)	0.0466 (5)	
H20A	−0.1732	−0.3570	0.1113	0.056*	
H20B	−0.1764	−0.2329	0.0556	0.056*	
C21	−0.32235 (19)	−0.2480 (3)	0.11024 (13)	0.0523 (6)	
H21A	−0.3451	−0.1499	0.1017	0.063*	
H21B	−0.3416	−0.2719	0.1582	0.063*	
C22	−0.3878 (2)	−0.3448 (3)	0.06021 (15)	0.0654 (7)	
H22A	−0.3655	−0.4430	0.0689	0.078*	
H22B	−0.3684	−0.3214	0.0123	0.078*	
C23	−0.5142 (2)	−0.3322 (4)	0.0676 (2)	0.1029 (13)	
H23A	−0.5328	−0.3385	0.1164	0.154*	
H23B	−0.5507	−0.4081	0.0420	0.154*	
H23C	−0.5392	−0.2419	0.0491	0.154*	
F1	−0.18395 (16)	0.0621 (2)	0.45430 (8)	0.0869 (6)	
N1	0.26695 (13)	0.06138 (18)	0.09789 (9)	0.0396 (4)	
N2	0.28234 (14)	0.0051 (2)	0.03381 (9)	0.0437 (5)	
N3	0.16543 (19)	−0.2048 (3)	−0.09756 (11)	0.0671 (6)	
N4	0.00947 (14)	−0.12733 (18)	0.08064 (9)	0.0386 (4)	
N5	0.01053 (13)	−0.02716 (18)	0.19646 (8)	0.0373 (4)	
N6	−0.13968 (15)	−0.1649 (2)	0.15346 (9)	0.0452 (5)	
H6A	−0.1566 (19)	−0.175 (3)	0.1970 (6)	0.054*	
O1	0.15378 (12)	0.11047 (17)	0.24170 (8)	0.0502 (4)	

### Atomic displacement parameters (Å^2^)

**Table:**

	*U*^11^	*U*^22^	*U*^33^	*U*^12^	*U*^13^	*U*^23^
C1	0.0649 (18)	0.080 (2)	0.087 (2)	−0.0194 (15)	−0.0151 (16)	−0.0128 (16)
C2	0.0469 (13)	0.0553 (14)	0.0527 (14)	−0.0113 (11)	−0.0035 (10)	0.0014 (11)
C3	0.0333 (12)	0.0704 (17)	0.0807 (19)	−0.0020 (11)	0.0045 (12)	−0.0152 (14)
C4	0.0386 (12)	0.0573 (15)	0.0698 (17)	−0.0003 (10)	0.0073 (11)	−0.0186 (12)
C5	0.0375 (11)	0.0428 (12)	0.0395 (12)	−0.0042 (9)	0.0018 (9)	0.0025 (9)
C6	0.0379 (11)	0.0435 (12)	0.0605 (14)	0.0020 (9)	0.0013 (10)	0.0014 (11)
C7	0.0520 (14)	0.0427 (13)	0.0628 (15)	−0.0041 (10)	0.0031 (11)	−0.0073 (11)
C8	0.0373 (11)	0.0505 (12)	0.0340 (11)	−0.0020 (9)	0.0000 (9)	0.0006 (9)
C9	0.0353 (10)	0.0389 (11)	0.0344 (11)	0.0006 (8)	−0.0018 (8)	0.0031 (9)
C10	0.0330 (10)	0.0376 (11)	0.0377 (11)	0.0011 (8)	0.0004 (8)	0.0013 (9)
C11	0.0417 (12)	0.0613 (14)	0.0375 (13)	−0.0070 (10)	0.0064 (9)	0.0005 (11)
C12	0.0357 (11)	0.0395 (11)	0.0359 (12)	−0.0004 (8)	−0.0031 (9)	0.0030 (9)
C13	0.0337 (10)	0.0394 (11)	0.0372 (11)	0.0023 (8)	−0.0025 (8)	−0.0008 (9)
C14	0.0347 (10)	0.0422 (11)	0.0365 (11)	−0.0046 (9)	0.0012 (8)	−0.0023 (9)
C15	0.0422 (12)	0.0484 (13)	0.0490 (14)	−0.0015 (10)	0.0025 (10)	0.0043 (10)
C16	0.0468 (13)	0.0523 (14)	0.0615 (16)	0.0020 (10)	0.0162 (11)	−0.0049 (12)
C17	0.0598 (15)	0.0617 (15)	0.0423 (14)	−0.0120 (12)	0.0157 (11)	−0.0092 (12)
C18	0.0569 (15)	0.0720 (16)	0.0376 (13)	−0.0013 (12)	0.0001 (10)	0.0037 (12)
C19	0.0466 (12)	0.0561 (14)	0.0418 (13)	0.0063 (10)	0.0014 (10)	0.0032 (10)
C20	0.0452 (12)	0.0517 (13)	0.0430 (13)	−0.0091 (10)	−0.0007 (10)	−0.0018 (10)
C21	0.0459 (13)	0.0547 (14)	0.0561 (15)	−0.0105 (11)	0.0009 (11)	−0.0026 (11)
C22	0.0543 (15)	0.0717 (18)	0.0700 (17)	−0.0164 (13)	−0.0053 (13)	−0.0101 (14)
C23	0.0517 (17)	0.104 (3)	0.152 (3)	−0.0189 (17)	−0.0184 (19)	−0.032 (2)
F1	0.0979 (12)	0.1106 (14)	0.0536 (10)	0.0002 (10)	0.0339 (9)	−0.0121 (9)
N1	0.0336 (9)	0.0448 (10)	0.0405 (10)	−0.0028 (7)	0.0036 (7)	−0.0026 (8)
N2	0.0391 (10)	0.0533 (11)	0.0390 (10)	−0.0034 (8)	0.0039 (8)	−0.0027 (8)
N3	0.0729 (15)	0.0862 (16)	0.0425 (12)	−0.0188 (12)	0.0066 (10)	−0.0109 (12)
N4	0.0366 (9)	0.0445 (10)	0.0346 (10)	−0.0043 (7)	−0.0011 (7)	0.0006 (7)
N5	0.0342 (9)	0.0461 (10)	0.0316 (9)	−0.0027 (7)	0.0016 (7)	−0.0008 (7)
N6	0.0430 (10)	0.0552 (11)	0.0374 (10)	−0.0145 (8)	0.0036 (8)	−0.0004 (9)
O1	0.0453 (9)	0.0611 (10)	0.0442 (9)	−0.0109 (7)	0.0015 (7)	−0.0139 (7)

### Geometric parameters (Å, °)

**Table:**

C1—C2	1.512 (3)	C14—C15	1.375 (3)
C1—H1A	0.9600	C14—C19	1.379 (3)
C1—H1B	0.9600	C14—N5	1.451 (3)
C1—H1C	0.9600	C15—C16	1.389 (3)
C2—C3	1.379 (3)	C15—H15	0.9300
C2—C7	1.384 (3)	C16—C17	1.364 (3)
C3—C4	1.382 (3)	C16—H16	0.9300
C3—H3	0.9300	C17—F1	1.353 (3)
C4—C5	1.372 (3)	C17—C18	1.366 (3)
C4—H4	0.9300	C18—C19	1.381 (3)
C5—C6	1.376 (3)	C18—H18	0.9300
C5—N1	1.434 (3)	C19—H19	0.9300
C6—C7	1.382 (3)	C20—N6	1.460 (3)
C6—H6	0.9300	C20—C21	1.503 (3)
C7—H7	0.9300	C20—H20A	0.9700
C8—N2	1.338 (3)	C20—H20B	0.9700
C8—C9	1.414 (3)	C21—C22	1.516 (3)
C8—C11	1.422 (3)	C21—H21A	0.9700
C9—N4	1.356 (3)	C21—H21B	0.9700
C9—C10	1.387 (3)	C22—C23	1.516 (4)
C10—N1	1.379 (2)	C22—H22A	0.9700
C10—C13	1.429 (3)	C22—H22B	0.9700
C11—N3	1.141 (3)	C23—H23A	0.9600
C12—N4	1.307 (3)	C23—H23B	0.9600
C12—N6	1.342 (3)	C23—H23C	0.9600
C12—N5	1.409 (3)	N1—N2	1.343 (2)
C13—O1	1.217 (2)	N6—H6A	0.861 (10)
C13—N5	1.416 (3)		
			
C2—C1—H1A	109.5	C17—C16—H16	120.5
C2—C1—H1B	109.5	C15—C16—H16	120.5
H1A—C1—H1B	109.5	F1—C17—C16	118.2 (2)
C2—C1—H1C	109.5	F1—C17—C18	119.1 (2)
H1A—C1—H1C	109.5	C16—C17—C18	122.6 (2)
H1B—C1—H1C	109.5	C17—C18—C19	118.3 (2)
C3—C2—C7	117.7 (2)	C17—C18—H18	120.8
C3—C2—C1	120.9 (2)	C19—C18—H18	120.8
C7—C2—C1	121.4 (2)	C14—C19—C18	120.2 (2)
C2—C3—C4	121.5 (2)	C14—C19—H19	119.9
C2—C3—H3	119.3	C18—C19—H19	119.9
C4—C3—H3	119.3	N6—C20—C21	110.36 (18)
C5—C4—C3	119.6 (2)	N6—C20—H20A	109.6
C5—C4—H4	120.2	C21—C20—H20A	109.6
C3—C4—H4	120.2	N6—C20—H20B	109.6
C4—C5—C6	120.5 (2)	C21—C20—H20B	109.6
C4—C5—N1	118.90 (19)	H20A—C20—H20B	108.1
C6—C5—N1	120.57 (18)	C20—C21—C22	113.5 (2)
C5—C6—C7	119.0 (2)	C20—C21—H21A	108.9
C5—C6—H6	120.5	C22—C21—H21A	108.9
C7—C6—H6	120.5	C20—C21—H21B	108.9
C6—C7—C2	121.8 (2)	C22—C21—H21B	108.9
C6—C7—H7	119.1	H21A—C21—H21B	107.7
C2—C7—H7	119.1	C21—C22—C23	113.0 (2)
N2—C8—C9	111.97 (18)	C21—C22—H22A	109.0
N2—C8—C11	120.53 (19)	C23—C22—H22A	109.0
C9—C8—C11	127.50 (19)	C21—C22—H22B	109.0
N4—C9—C10	126.10 (19)	C23—C22—H22B	109.0
N4—C9—C8	129.92 (18)	H22A—C22—H22B	107.8
C10—C9—C8	103.89 (17)	C22—C23—H23A	109.5
N1—C10—C9	107.14 (17)	C22—C23—H23B	109.5
N1—C10—C13	132.08 (18)	H23A—C23—H23B	109.5
C9—C10—C13	120.51 (18)	C22—C23—H23C	109.5
N3—C11—C8	179.1 (3)	H23A—C23—H23C	109.5
N4—C12—N6	120.35 (18)	H23B—C23—H23C	109.5
N4—C12—N5	123.46 (17)	N2—N1—C10	111.51 (16)
N6—C12—N5	116.17 (18)	N2—N1—C5	118.25 (16)
O1—C13—N5	120.49 (18)	C10—N1—C5	130.19 (17)
O1—C13—C10	128.07 (19)	C8—N2—N1	105.48 (16)
N5—C13—C10	111.42 (16)	C12—N4—C9	114.82 (17)
C15—C14—C19	120.5 (2)	C12—N5—C13	123.64 (16)
C15—C14—N5	120.46 (18)	C12—N5—C14	121.19 (16)
C19—C14—N5	118.84 (18)	C13—N5—C14	115.17 (15)
C14—C15—C16	119.4 (2)	C12—N6—C20	122.23 (18)
C14—C15—H15	120.3	C12—N6—H6A	116.0 (16)
C16—C15—H15	120.3	C20—N6—H6A	116.6 (16)
C17—C16—C15	118.9 (2)		
			
C7—C2—C3—C4	−0.4 (4)	N6—C20—C21—C22	−179.1 (2)
C1—C2—C3—C4	179.6 (3)	C20—C21—C22—C23	−179.7 (3)
C2—C3—C4—C5	−0.3 (4)	C9—C10—N1—N2	−0.6 (2)
C3—C4—C5—C6	1.3 (4)	C13—C10—N1—N2	173.4 (2)
C3—C4—C5—N1	−179.5 (2)	C9—C10—N1—C5	−178.14 (19)
C4—C5—C6—C7	−1.7 (3)	C13—C10—N1—C5	−4.2 (4)
N1—C5—C6—C7	179.2 (2)	C4—C5—N1—N2	−38.9 (3)
C5—C6—C7—C2	1.0 (4)	C6—C5—N1—N2	140.2 (2)
C3—C2—C7—C6	0.0 (4)	C4—C5—N1—C10	138.4 (2)
C1—C2—C7—C6	−180.0 (2)	C6—C5—N1—C10	−42.4 (3)
N2—C8—C9—N4	−177.1 (2)	C9—C8—N2—N1	0.0 (2)
C11—C8—C9—N4	3.1 (4)	C11—C8—N2—N1	179.83 (19)
N2—C8—C9—C10	−0.3 (2)	C10—N1—N2—C8	0.4 (2)
C11—C8—C9—C10	179.8 (2)	C5—N1—N2—C8	178.24 (17)
N4—C9—C10—N1	177.48 (18)	N6—C12—N4—C9	178.61 (18)
C8—C9—C10—N1	0.6 (2)	N5—C12—N4—C9	0.1 (3)
N4—C9—C10—C13	2.7 (3)	C10—C9—N4—C12	−1.8 (3)
C8—C9—C10—C13	−174.26 (18)	C8—C9—N4—C12	174.3 (2)
N2—C8—C11—N3	159 (18)	N4—C12—N5—C13	0.7 (3)
C9—C8—C11—N3	−21 (18)	N6—C12—N5—C13	−177.79 (18)
N1—C10—C13—O1	3.8 (4)	N4—C12—N5—C14	179.94 (18)
C9—C10—C13—O1	177.1 (2)	N6—C12—N5—C14	1.4 (3)
N1—C10—C13—N5	−174.8 (2)	O1—C13—N5—C12	−178.73 (18)
C9—C10—C13—N5	−1.5 (3)	C10—C13—N5—C12	0.0 (3)
C19—C14—C15—C16	−0.5 (3)	O1—C13—N5—C14	2.0 (3)
N5—C14—C15—C16	−175.46 (19)	C10—C13—N5—C14	−179.23 (16)
C14—C15—C16—C17	1.2 (3)	C15—C14—N5—C12	−78.0 (2)
C15—C16—C17—F1	178.7 (2)	C19—C14—N5—C12	106.9 (2)
C15—C16—C17—C18	−1.2 (4)	C15—C14—N5—C13	101.2 (2)
F1—C17—C18—C19	−179.4 (2)	C19—C14—N5—C13	−73.8 (2)
C16—C17—C18—C19	0.4 (4)	N4—C12—N6—C20	5.5 (3)
C15—C14—C19—C18	−0.3 (3)	N5—C12—N6—C20	−175.88 (18)
N5—C14—C19—C18	174.8 (2)	C21—C20—N6—C12	−152.0 (2)
C17—C18—C19—C14	0.3 (4)		

### Hydrogen-bond geometry (Å, °)

**Table:**

*D*—H···*A*	*D*—H	H···*A*	*D*···*A*	*D*—H···*A*
N6—H6A···O1^i^	0.861 (10)	2.32 (2)	2.904 (2)	125 (2)
C15—H15···N3^ii^	0.93	2.58	3.449 (3)	155
C19—H19···N3^iii^	0.93	2.50	3.219 (3)	134
C6—H6···Cg1^iv^	0.93	2.82	3.671 (2)	152

Symmetry codes: (i) −*x*, *y*−1/2, −*z*+1/2; (ii) −*x*, −*y*, −*z*; (iii) *x*, −*y*−1/2, *z*+1/2; (iv) −*x*, *y*+1/2, −*z*+1/2.
